# Assessing the quality of *Smilacis Glabrae Rhizoma* (*Tufuling*) by colormetrics and UPLC-Q-TOF-MS

**DOI:** 10.1186/s13020-016-0104-y

**Published:** 2016-07-06

**Authors:** Xicheng He, Tao Yi, Yina Tang, Jun Xu, Jianye Zhang, Yazhou Zhang, Lisha Dong, Hubiao Chen

**Affiliations:** School of Chinese Medicine, Hong Kong Baptist Univesity, Hong Kong, China; School of Pharmacy, Gui Yang Collage of Traditional Chinese Medicine, Guiyang, 550002 China

**Keywords:** *Smilacis Glabrae Rhizoma*, Colormetrics, Fingerprint analysis

## Abstract

**Background:**

The quality of the materials used in Chinese medicine (CM) is generally assessed based on an analysis of their chemical components (e.g., chromatographic fingerprint analysis). However, there is a growing interest in the use of color metrics as an indicator of quality in CM. The aim of this study was to investigate the accuracy and feasibility of using color metrics and chemical fingerprint analysis to determine the quality of *Smilacis Glabrae Rhizoma* (*Tufuling*) (SGR). The SGR samples were divided into two categories based on their cross-sectional coloration, including red SGR (R-SGR) and white SGR (W-SGR).

**Methods:**

Forty-three samples of SGR were collected and their colors were quantized based on an RGB color model using the Photoshop software. An ultra-performance liquid chromatography/quadrupole time-of-flight mass spectrometry (UPLC/QTOF MS) system was used for chromatographic fingerprint analysis to evaluate the quality of the different SGR samples. Hierarchical cluster analysis and dimensional reduction were used to evaluate the data generated from the different samples. Pearson correlation coefficient was used to evaluate the relationship between the color metrics and the chemical compositions of R-SGR and W-SGR.

**Results:**

The SGR samples were divided into two different groups based on their cross-sectional color, including color A (CLA) and B (CLB), as well as being into two separate classes based on their chemical composition, including chemical A (CHA) and B (CHB). Standard fingerprint chromatograms were for CHA and CHB. Statistical analysis revealed a significant correlation (*Pearson’s r* = −*0.769, P* < *0.001*) between the color metrics and the results of the chemical fingerprint analysis.

**Conclusions:**

The SGR samples were divided into two major clusters, and the variations in the colors of these samples reflected differences in the quality of the SGR material. Furthermore, we observed a statistically significant correlation between the color metrics and the quality of the SGR material.

## Background

The quality of Chinese medicine (CM) continues to attract considerable attention. The quality control procedures traditionally used in CM are based on the color, smell and texture characteristics of the raw materials, as well as several other sensory properties. Color in particular is an essential method of quality control for evaluating the materials used in CM, but the effectiveness of this quality indictor is limited by its subjective nature. However, recent technological advances mean that it is now possible to measure and quantify colors using instrumental methods. For example, the major colors of an image can be represented by 8 bits in a computer, meaning that each color axis can be represented by 8 bits or 2^8^ = 256 different values in the Red–Green–Blue (RGB) color space [1, 2]. Using imaging software, it is possible to read the RGB values of every pixel of a sample image. These data can subsequently be used to generate average RGB values for the different areas of an image, which can be used to represent color information.

Today, the quality of CM is mainly determined based on an analysis of their chemical composition using sophisticated analytical techniques, such as chromatographic fingerprint analysis, which can be used to provide an indication of their intrinsic quality. Instrument-based analytical techniques have therefore been widely accepted for quality evaluation and species identification in CM, supplementing traditional techniques based on the sensory properties of these materials [3–5]. Establishing a connection between the color and the chemical composition of the materials used in CM would provide solid evidence for establishing a new method of quality control, which could validate the correlation between the sensory properties and quality of CM. In this study, we have selected *Smilacis Glabrae Rhizoma* (*Tufuling*) (SGR) as a model medicinal herb to evaluate the relationship between the quality of this material and its color metrics and chemical composition data.

**Fig. 1:**
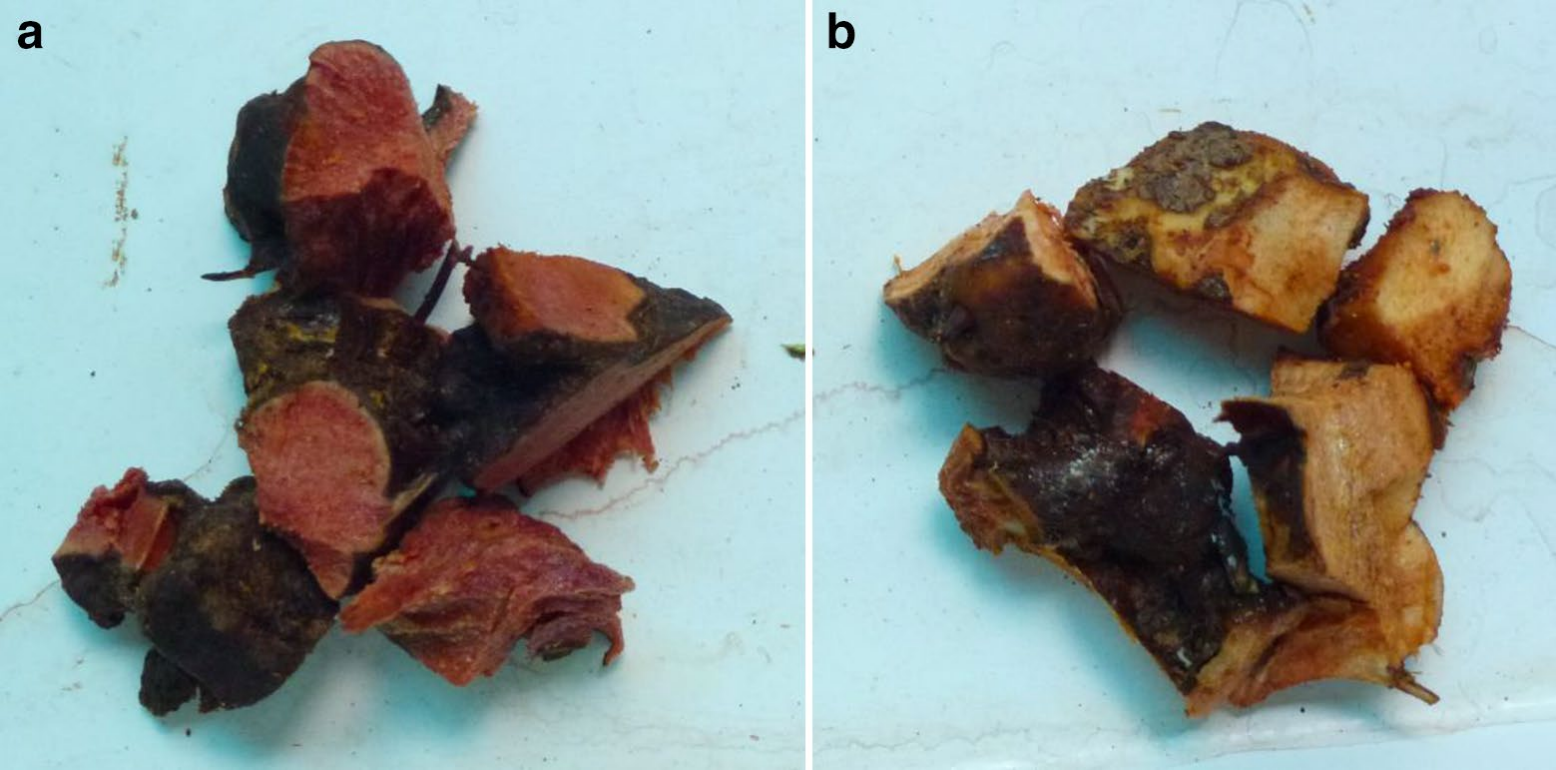
The samples of SGR (**a**) Red cross-sectional SGR. **b** White cross-sectional SGR

SGR, which is the dried rhizome of *Smilax glabra* Roxb., can be red or white (Fig. 1). Generally, the therapeutic effects of white SGR are believed to be more pronounced than those of the red material [6–8], suggesting that the color of this material could be used as an indicator of its quality. SGR samples can therefore be divided into two groups depending on their cross-sectional color, including red SGR (R-SGR) and white SGR (W-SGR) [6].

However, the results of another study revealed that most SGR samples contain both red and white cross-sections, and that the quality of these materials can vary considerably [9]. Despite the potential implications of this discovery, there has been other work conducted in this area, and the quality of SGR is currently evaluated by chemical composition analysis with no regard for differences in the apparent color.

**Fig. 2:**
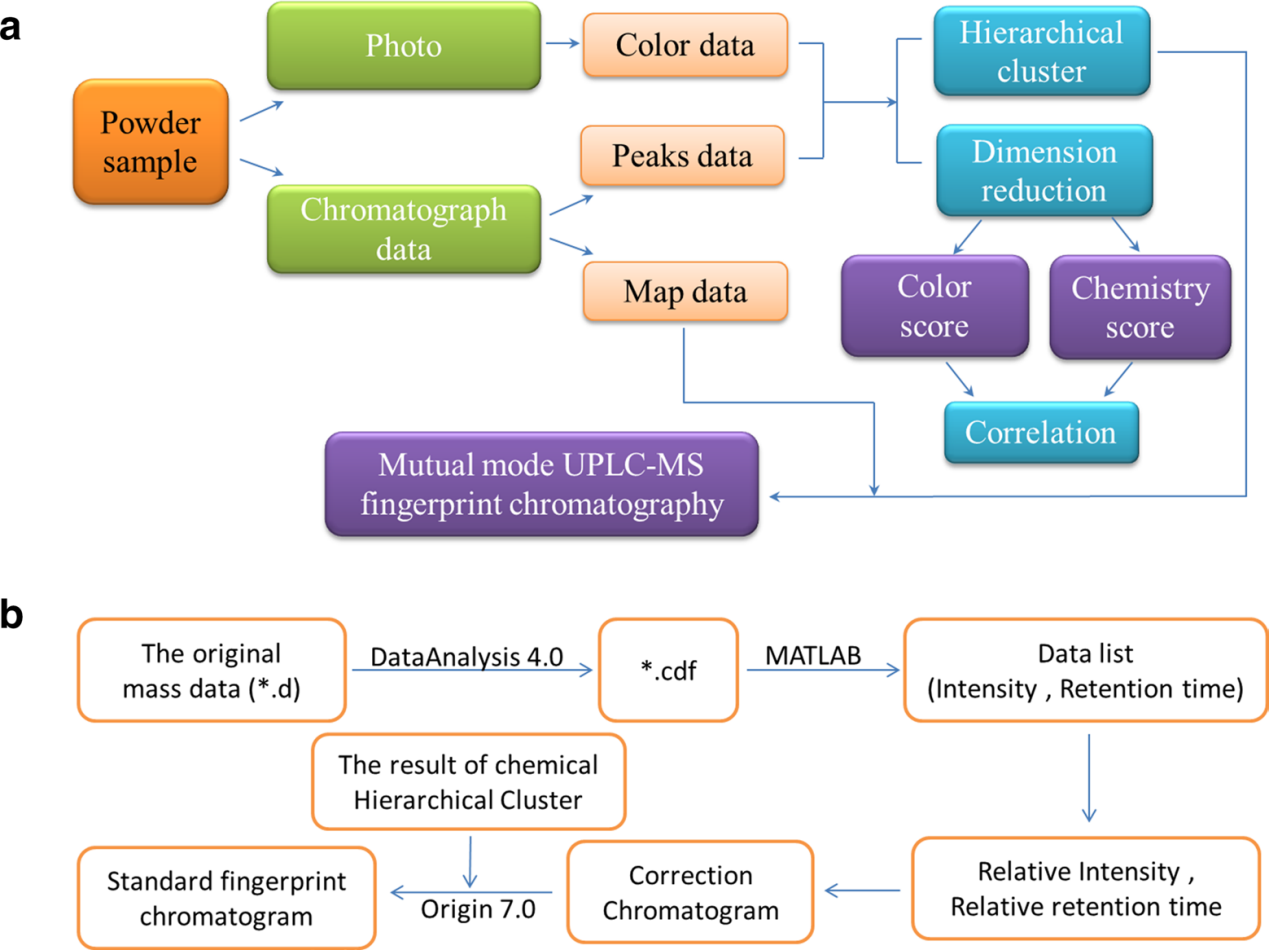
Flow chart of this work. **a** Flow chart of the experiment design process (**b**) Flow chart for establishing a standard fingerprint chromatogram $R{\text{Intens}} .= \frac{{{\text{Intens}} .}}{{{\text{SPH}} \times {\text{W}}}},\quad R{\text{RT }} = \frac{\text{RT}}{\text{SRT}},$ where *R*Intens is the relative Intensity, *R*RT is the relative retention time, SPH and SRT are the peak height and retention time, respectively, of the internal standard (IS), and W was sample weight (g)

This aim of this study was to investigate the accuracy and feasibility of using color metrics and chemical fingerprint analysis to analyze the quality of SGR. In this study, we used image software to quantify the color information of the red and white SGR samples based on color data generated using an RGB model. We also used ultra-performance liquid chromatography/quadrupole time-of-flight mass spectrometry (UPLC/QTOF MS) to produce chemical chromatographic fingerprints for these different samples. Hierarchical analysis and dimensional reduction methods were used to generate color and chemical scores for the different SGR samples, and standard fingerprint chromatograms were established according to the clustering results of the main model. Pearson correlation coefficient was used to evaluate the relationship between the color and the chemical composition of the different samples, which revealed a statistically significant relationship between these two variables (Fig. 2a).

## Methods

### Materials

Information pertaining to the different SGR samples is shown in Table 1. All of these samples were authenticated as the genuine rhizome of *S. Glabra* by Prof. Lisha Dong (School of Pharmacy, Gui Yang College of Traditional Chinese Medicine, China). These samples were authenticated based on a comparison of their flowers with those of *Smilax Glabra* Roxb., according to the Flora of China [10].

**Table 1:** Details of the different SGR samples

No.	Batch no.	Location	Color of cross-section
1	20080728	Guiyang, Guizhou	Red
2	20090403	Guiyang, Guizhou	Red
3	20090828	Taizhou, Zhejiang	White
4^a^	20090922	Beijing Tongrentang	White
5^a^	20090928	Luoyang, Henan	White
6	20100316	Guangdong	White
7	20100410	Guizhou	White
8^a^	20100429	Sichuan	White
9	20100513	Liubanshui, Guizhou	Red
10	20100531	Guiyang, Guizhou	Red
11	20100606	Guiyang, Guizhou	White
12	20100704	Anshun, Guizhou	Red
13^a^	20100714	Anhui	White
14	20100715	Guiyang, Guizhou	Red
15	20100821	Guiyang, Guizhou	Red
16	20100826	Guiyang, Guizhou	White
17	20100901	Guiyang, Guizhou	White
18	20100924	Guiyang, Guizhou	Red
19	20101028	Tongren, Guizhou	White
20	20100104	Zhijin, Guizhou	Red
21	20101005	Duyun, Guizhou	Red
22	20101111	Zunyi, Guizhou	White
23^a^	101201	Guiyang Tongrentang	White
24	20110210	Zhijin, Guizhou	Red
25^a^	110410	Guiyang Tongrentang	White
26	20110715	Duyun, Guizhou	Red
27^a^	20110601	Anhui	White
28	20110805	Guiyang, Guizhou	Red
29	20100812	Guiyang, Guizhou	Red
30	20110831	Guiyang, Guizhou	Red
31	20110903	Guiyang, Guizhou	Red
32^a^	20111005	Hangzhou, Zhejiang	White
33	20110318	Puan, Guizhou	Red
34	20120415	Hezhang, Guizhou	Red
35	20120714	Ceheng, Guizhou	White
36	20130104	Hongkong	White
37	20120402	Puan, Guizhou	Red
38	20120418	Duyun, Guizhou	White
39	20121220	Hong Kong	White
40	20130214	Qingyuan, Guangdong	White
41	20130530	Baise, Guangxi	Red
42	20130626	Nanning, Guangxi	White
43	20130628	Wuzhou, Guangxi	White

^a^Represent purchased samples

Analytical grade methanol (Labscan, Bangkok, Thailand) was used to prepare the reference standards, as well as the samples of the different extracts. Chromatography-grade formic acid (Fluka, Buchs, Switzerland), chromatography-grade acetonitrile (Labscan, Bangkok, Thailand) and Milli-Q water (Millipore, Bedford, MA, USA) were used to prepare the mobile phases for chromatography. Analytical standards of astilbin, engeletin, (+)-catechin, (–)-epicatechin and 7,4′-dihydroxyflavone were obtained from the National Institute for the Control of Pharmaceutical and Biological Products, China. All of these chemical standards were greater than 98 % in purity.

### Analytical procedures

#### Color analysis

**Fig. 3:**
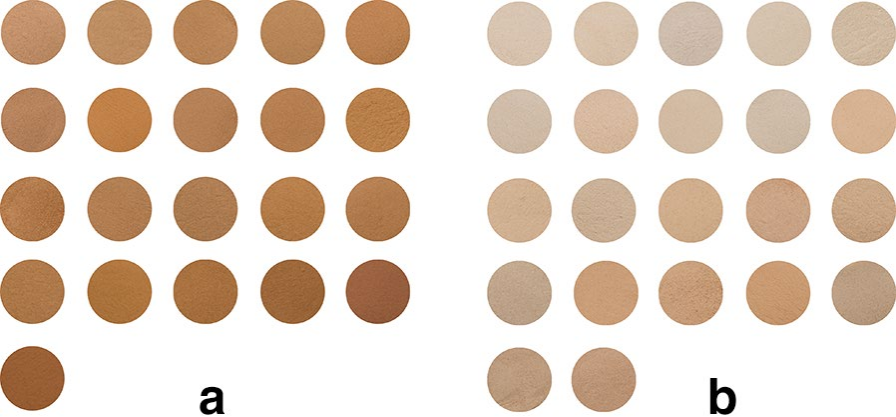
Photos of the 43 SGR powders. **a** Red cross-sectional SGR. **b** White cross-sectional SGR

The SGR samples were pulverized, and the resulting powders were sieved through a 120-mesh screen. The sieved powders were then stored in the dark prior to being analyzed. Samples of the sieved powders were uniformly dispersed on the surface of a glass culture dish (diameter, 60 mm) which was placed in a Desktop Proeasy LTM-101 light source system (Nexor, Guangzhou, China) together with a standard color checker (Mennon, Beijing, China) and an 18 % Grey Card (Topimage, Taipei, Taiwan). Photographic images were recorded using a Nikon D5200 camera with a focal length of 24 mm (M grade, A:F8, S:1/40). The photographic images were visualized using the Adobe Photoshop CS5 software (Adobe, San Jose, CA, USA). The Grey Card was used to correct the white balance in the images using the gray-pipette tool from Photoshop, whereas the standard color checker was used to produce a file containing less than five color errors. Five different areas of each photograph were selected for sampling, with each sampling area set as 51 × 51 pixels. Color data were obtained by calculating the average values of the RGB data, which were measured using the color information derived from each sample with the eyedropper tool from Photoshop (Fig. 3).

Color data were subjected to partial least squares (PLS) regression analysis using the MATLAB software (MathWorks, Natick, MA, USA) to analyze the relationship between the colors in R-SGR and W-SGR, as well as quantifying the boundary between R-SGR and W-SGR. The dependent variable used in this model was G, whereas R and B were used as independent variables.

#### Chemical analysis

An SGR power sample of 0.5 g was added to 20.0 mL of 60 % (v/v) methanol in water, and the resulting mixture was sonicated (CP1800 HT, Crest, Penang-, Malaysia) for 30 min at room temperature. The sonicated mixture was centrifuged (5810, Eppendorf, Hamburg, Germany) at 2100×*g* for 5 min at room temperature, and 400 µL of the supernatant was placed in a volumetric flask, followed by 100 µL of 7,4′-dihydroxyflavone (0.1249 mg/mL), which was added as an internal standard (IS). The total volume of the mixture in the volumetric flask was then adjusted to 10 mL by the addition of methanol, and the resulting sample solution was filtered through a 0.22-µm filter before being analyzed by LC-MS. Two replicates of each SGR sample were prepared and analyzed in the same way. All of the extracts were stored at 4 °C before use.

UPLC analysis was performed on a Waters Acquity system (Waters Corporation, Milford, MA, USA) equipped with a quaternary solvent manager and a sample manager. This system was also coupled to a Micromass QTOF premier mass spectrometer (Bruker Daltonics, Milford, MA, USA) equipped with an electrospray ionization (ESI) interface. Chromatographic separation was conducted over a Waters C18 T3 column (1.8 µm, 2.1 × 100 mm) eluting with a mobile phase consisting of 0.1 % formic acid in water (A) and 0.1 % formic acid in acetonitrile (B) with linear gradient elution at a flow rate of 0.35 mL/min. The gradient elution was conducted as follows: 5–14 % B (0–1.5 min), 14–16 % B (1.5–6.0 min), 16–20 % B (6.0–20.0 min), 20–100 % B (20.0–30.0 min). The column temperature was kept at 40 °C throughout the entire process. The sample size for injection into the UPLC system was set at 2 µL. The ESI source of the MS was connected to the UPLC system via a capillary through the UV cell outlet. MS data were collected with a capillary voltage of 3.5 kV in the negative ionization mode. Nitrogen was used as a desolvation gas at a flow rate was of 8 L/h. The scan range (*m/z*) was set at 100–1000 Da.

Extracted ion chromatograms (EIC) were used to obtain the peak areas of the different components, and the relative peak areas were then calculated based on the peak area of the IS. The relative peak areas were calculated using the following equation:

$$ RPA_{i} = \frac{{A_{i} }}{{A_{S} \times W}}, $$

where *RPA* is the relative peak area, *A* is the peak area of a specific component, *A*_*s*_ is the peak area of the IS, W is the sample weight and *i* is the peak number.

The original data were saved as CDF files using version 4.0 of the DataAnalysis software (Bruker, USA). The MATlab R2012b software (MathWorks, USA) was then used to extract information from the CDF files, including retention time (RT) and intensity (Intens.) data (Fig. 2b). The scan range (*m/z*) was set at 100–800 Da. According to the result of chemical hierarchical cluster, chromatogram maps corrected by IS were combined to generate standard chromatogram by mean value method.

The correlation coefficient, *r*_*con*_, was used in the current study to assess the consistency of the chemical composition of SGR using the following equations [3, 11, 12]:

$$ r_{con} = \tfrac{{\sum\nolimits_{i = 1}^{num} {x_{i} y_{i} } }}{{\sqrt {\left( {\sum\nolimits_{i = 1}^{num} {\left( {x_{i} } \right)^{2} } } \right)\left( {\sum\nolimits_{i = 1}^{num} {\left( {y_{i} } \right)^{2} } } \right)} }}, $$

$$ \bar{x} = \left( {\sum\limits_{i = 1}^{num} {x_{i} } } \right)/n,\quad \bar{y} = \left( {\sum\limits_{i = 1}^{num} {y_{i} } } \right)/n, $$

where *x*_*i*_ and *y*_*i*_ are the *i*th elements in two different fingerprints (i.e., *x* and *y*), *num* is the number of the elements in the fingerprints and $\bar{x}$ and $\bar{y}$ are the mean values of the *num* elements in fingerprints *x* and *y*, respectively.

### Correlation of color metrics and chemical fingerprint

Dimensional reduction was used to convert complex chemical information and color data into one or a few simple variables. Factor analysis was used to reduce the number of dimensions and extract the most important information from the original data. To verify that the resulting data sets were suitable for factor analysis, we checked that the Kaiser–Meyer–Olkin (KMO) values were greater than 0.6 and that the Bartlett’s test of Sphericity value was significant (significant values should be less than 0.05). The color and chemical scores were subsequently calculated using factor analysis, and the results were used to evaluate the correlation between the color metrics and the chemical fingerprint by Pearson correlation coefficient.

### Statistical analysis

#### Hierarchical clustering

Hierarchical clustering was conducted using version 20.0 of the IBM SPSS software (IBM, Armonk, NY, USA) to allow for the standardization of the color and chemical data, followed by hierarchical clustering by classification analysis.

#### Dimensional reduction

Factor analysis was used for the dimensional reduction of the color data obtained for the different SGR samples, and the resulting factor score was subsequently regarded as the color score. Factor analysis was used for dimensional reduction processing, and the principal components were considered to be independent based on the factor rotation method. Based on the different values of the principal component analysis, we calculated a cumulative score for the chemical data. *P* value less than 0.05 were considered statistically significant.

#### Correlation analysis

The relationship between the color and chemical scores was evaluated using Pearson correlation coefficient. A scatter plot of these data was then generated using version 20.0 of the SPSS software with the chemical and color scores on the x and y axes, respectively. *P* value less than 0.05 were considered statistically significant.

## Results and discussion

### Color analysis

**Fig. 4:**
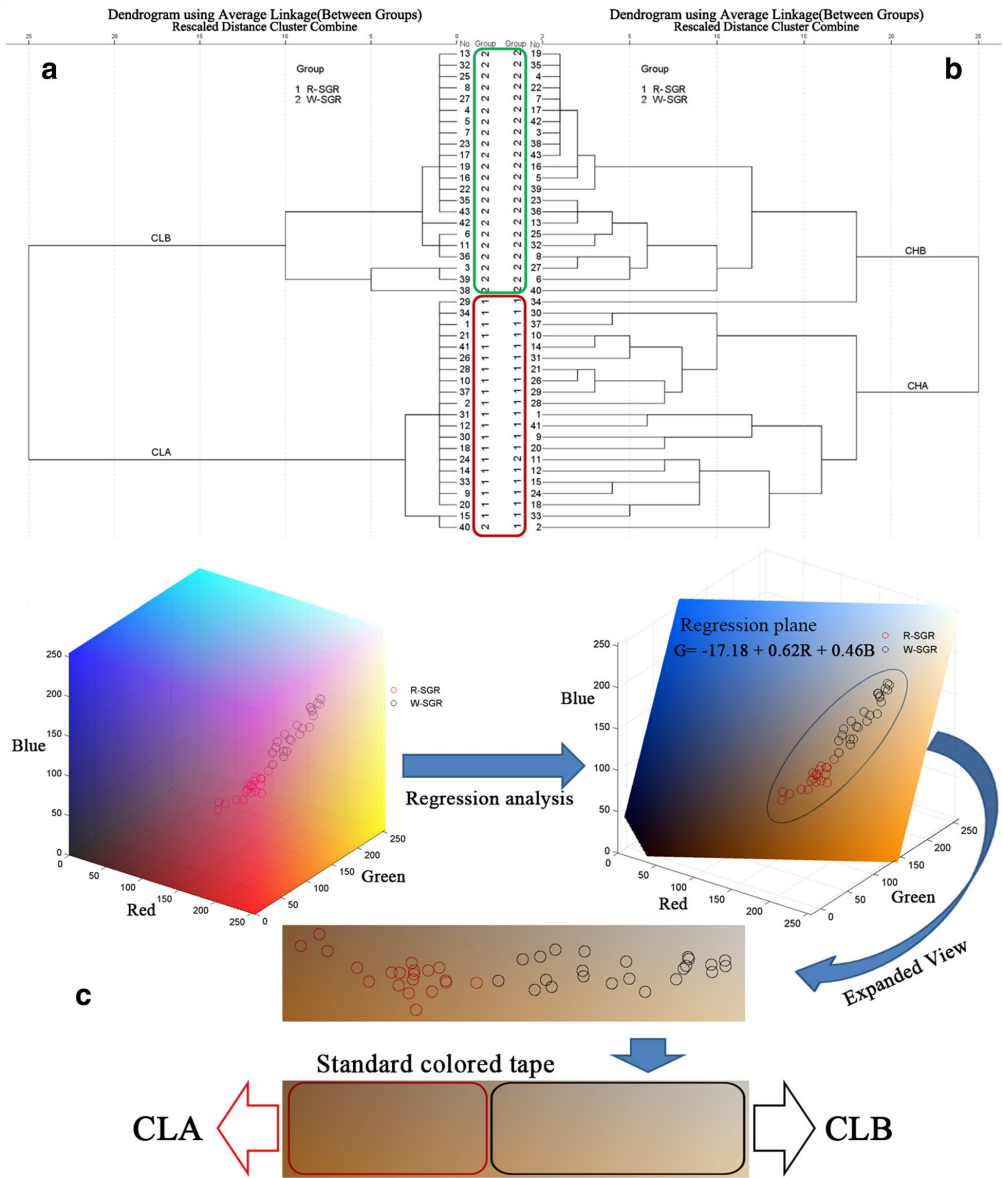
Division of the samples into two classes using hierarchical analysis; CLA and CLB, based on color; and CHA and CHB, based on chemical composition. A multiple regression model was established in color space based on our regression analysis of the color data. **a** Cluster dendrogram of the different colors. **b** Cluster dendrogram constructed from the EIC peak areas of the 43 SGR samples. **c** Regression model of SGR into the RGB color space and the standard tape of the SGR samples

A color dendrogram of the 43 different SGR samples evaluated in this study is shown in Fig. 4a. This figure shows that the SGR samples were divided into two major clusters (i.e., R-SGR and W-SGR) based on their color. The R-SGR samples were grouped into one cluster defined as “CLA”, whereas the W-SGR samples were grouped into another cluster defined as “CLB”. The results of quantitative color analysis supported the result of this subjective classification, suggesting that SGR samples were composed of both white and red materials. However, it is noteworthy that all of these samples were defined as being the same based on their original botanical identification.

The major colors of the different SGR samples were represented by one of 256 different values in the RGB color space. Based on the results of the regression analysis, we established a multiple regression model in color space using the following regression equation (Fig. 4c):

$$ G = -17.18 + 0.62R + 0.46B \,(R^2 = 0.998;  P < 0.001).$$

All of the R-SGR and W-SGR samples were represented in this regression model. As shown in the expanded view, this model established a standard tape of SGR, with R-SGR and W-SGR on either side based on the color location, allowing for the two clusters to be differentiated into two different zones.

### Chemical analysis

The 43 SGR samples were also subjected to chemical analysis and separated into two categories based on the results by hierarchical clustering (Fig. 4b). The R-SGR samples were grouped in one cluster, which was defined as “CHA”, whereas the W-SGR samples were grouped in anther cluster, which was defined as “CHB”, indicating that there were two different types of SGR based on differences in their chemical composition.

**Fig. 5:**
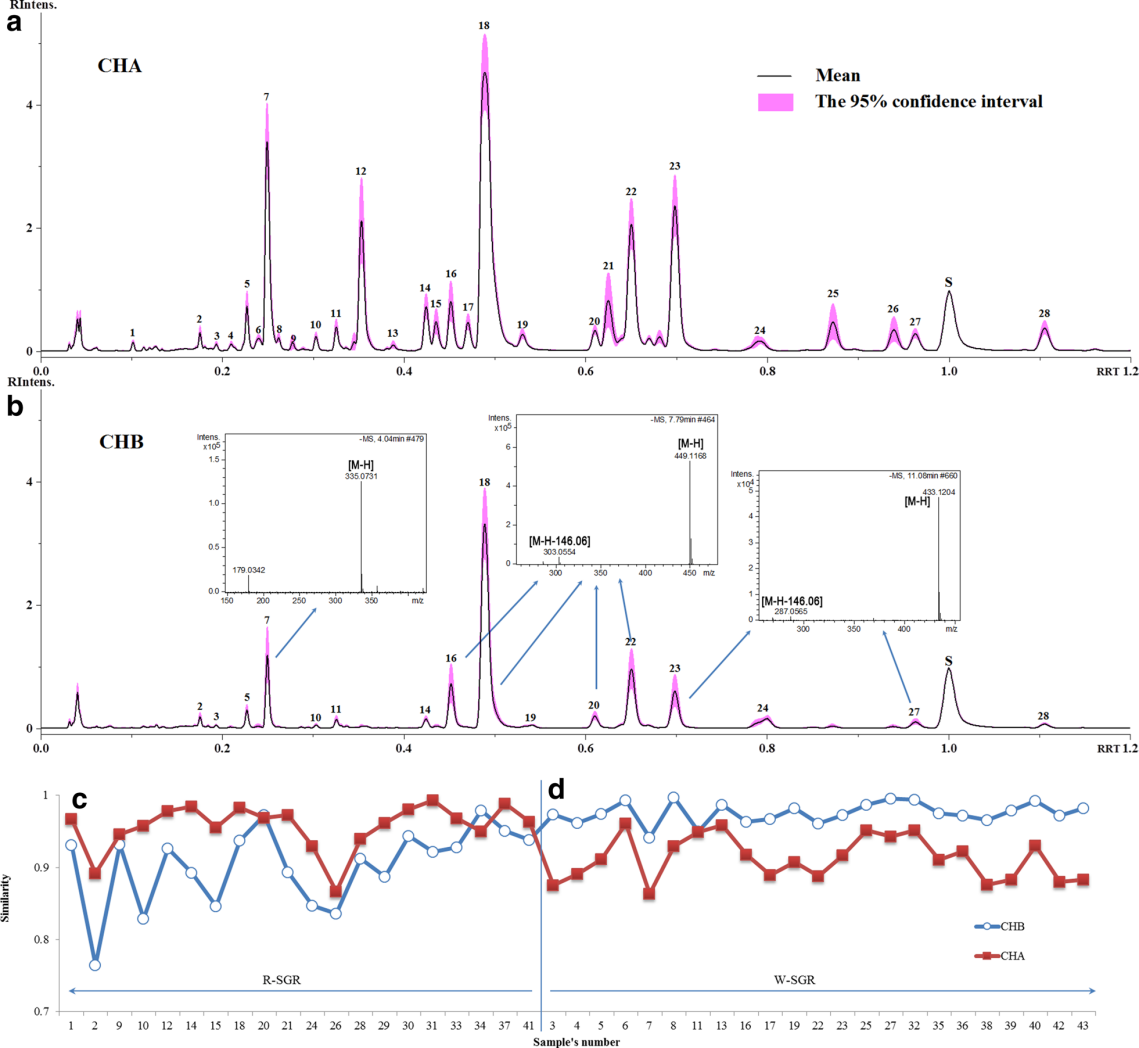
Standard fingerprint chromatographs and mass spectra of the CHA and CHB samples collected by UPLC/QTOF MS (**a**) Standard fingerprint chromatographs for CHA showing 95 % confidence intervals. **b** Standard fingerprint chromatographs for CHB showing 95 % confidence intervals (the 95 % confidence intervals resulted in increased precision of the standard fingerprint chromatographs). **c** The correlation coefficients of CHA. **d** The correlation coefficients of CHB (the *red* and *blue lines* represent the correlation coefficients of CHA and CHB, respectively). * indicates that the peak was identified based on a comparison with the literature [13–15]

The chemical compositions in the extracts of R-SGR and W-SGR were analyzed by UPLC/QTOF MS. Twenty-eight characteristic peaks were found in the EICs and nine compounds were identified based on their fragmentation patterns and a comparison of their MS data with those available in the literature (Table 2) [13, 14, 15]. The R-SGR and W-SGR samples contained different numbers of peaks (compositions), as shown in Fig. 5a, b. The amounts of the different components also changed between the two different sample types.

**Table 2:** Information concerning the different compounds found in the chromatogram of SGR

No.	RRT	m/z (–)	Compounds
1	0.106	323.13	
2	0.174	289.07	(+)-Catechin
3	0.180	561.14	
4	0.211	561.14	
5	0.230	289.07	(−)-Epicatechin
6	0.236	561.14	
7*	0.255	335.07/179.04	5-O-Caffeoylshikimic acid
8	0.261	561.14	
9	0.280	465.10	
10	0.304	339.07	
11	0.329	335.07/179.04	
12	0.354	449.08/269.05	
13	0.391	449.08	
14	0.422	451.10/341.06	
15	0.435	507.11	
16*	0.453	449.08/303.05	Neoastilbin
17	0.472	449.08/269.05	
18	0.491	449.08/303.05	
19	0.534	723.17	
20*	0.609	449.08/303.05	Astilbin
21	0.634	491.11	
22*	0.652	449.08/303.05	Isoastilbin
23	0.702	433.1/269.04	Engeletin
24	0.795	723.50	
25	0.876	491.11	
26	0.944	491.11	
27*	0.969	433.11/269.04	Isoengeletin
IS	1.000	253.05	7,4′-di Hydroxy flavone
28	1.106	451.10	

*Represent that the peak was identified by references

The precision of this analytical method was determined by injecting the same sample solution into the system on six consecutive occasions. The peak areas of the different components were then taken as measures of the precision and expressed as relative standard deviation (RSD) values, which resulted in a precision of 2.97 %. Six peaks with peak areas greater than 3 % and relative retention time (RRT) of 0.25, 0.35, 0.45, 0.49, 0.65 and 0.70 were chosen to calculate the repeatability and stability of the method. The RSD of RT repeatability was less than 0.24 %, whereas the relative peak area (RPA) repeatability was less than 4.72 %, which showed good stability over 8 h at less than 4.57 %.

Standard fingerprint chromatograms of samples belonging to the CHA and CHB groups were established based on the result of the main clustering model (Fig. 5a, b), and calculated with a 95 % confidence interval. A pronounced difference was observed between the two modes of examination used in the standard fingerprint chromatograms. The standard fingerprint chromatograms of CHA contained 28 peaks, whereas standard fingerprint chromatograms of CHB contained only 17 peaks without peak numbers 12, 17, 21, 25 and 26. Moreover, the amount of the different components varied considerably between CHA and CHB. For example, the peak corresponding to astilbin (peak 18), which was the biggest of all of the peaks detected in CHA and CHB, was much smaller in CHB than it was in CHA. Five other peaks, including peaks 2, 5, 7, 22 and 23, also appeared at much lower levels in the standard fingerprint in CHB.

The relationships between the fingerprint chromatograms could be analyzed by comparing the similarities between specific reference points. The correlation coefficient was used in this study to examine the similarities between the samples clustered into the CHA and CHB groups. The correlation coefficients of the CHA were more than 0.9 (the red line in Fig. 5c), indicating that the standard fingerprint chromatograms represented the chemical characteristics of R-SGR. All of the correlation coefficients of the CHB were greater than 0.93 (the blue line in Fig. 5d), and the similarities in this group were higher than those of the CHA group, indicating that the standard fingerprint chromatograms represented the chemical characteristics of W-SGR. The standard fingerprint chromatograms of CHA and CHB were stable and consistent.

### Correlation of the color metrics and chemical fingerprint data

For this example, we obtained a KMO value of 0.656, and the results of a Bartlett’s test revealed that this result was significant (*P* < 0.001), indicating that the use of factor analysis was appropriate [16]. The three variables (RGB) for the color data were transformed into one factor score, affording a total cumulative variance of 97.851 %. The color score was calculated as follows:

$$ Color \, score \, = \, ZR\, \times \,0. 3 3 4\, + \,\, \, ZG\,\, \times \,0. 3 40\, + \,ZB \, \times \,0. 3 3 7, $$

where *ZR, ZG* and *ZB* are standardized data for *R, G* and *B,* respectively. The constants were defined as the component score coefficients. A high color score was indicative of white SGR, whereas a low color score was indicative of red SGR.

The results of the chemical data led to the identification of six principal factors based on 28 peaks by factor analysis. Principal component analysis was then used to translate these six variables into a chemical score, which gave a total cumulative variance of 88.298 % together with a KMO value of 0.639. Furthermore, Bartlett’s test was significant (*P* < 0.001). The formula used to compute the chemical score was as follows: Chemistry score = FAS1 × 20.947 % + FAS2 × 16.818 % + FAS3 × 16.702 % + FAS4 × 15.418 % + FAS5 × 13.717 % + FAS6 × 4.696 %.

FAS was used as a factor score, with constant squared loadings for each factor. This method allowed for the evaluation of increasingly complex factors, and also provided a novel strategy for evaluating quality.

**Fig. 6:**
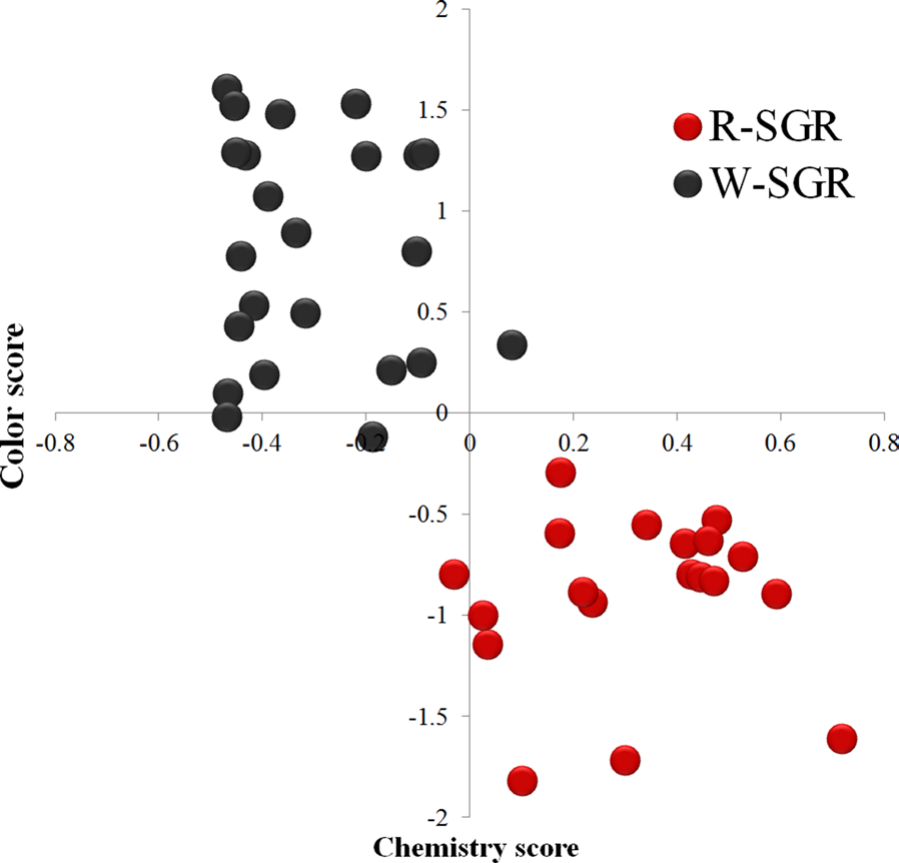
Scatter diagram of the color and chemistry scores

The Pearson correlation coefficient was determined to be −0.769 (double side test *P* < 0.001), suggesting that there was a significant correlation between the color and chemistry scores (Fig. 6).

The result of the dimensional reduction and correlation analysis indicated that there was a statistically significant relationship between the colors and chemical components of the different SGR samples, as did the results of the hierarchical clustering. These results therefore suggest that it is feasible to use color (red and white) to distinguish between two different qualities or specifications of SGR.

## Conclusions

SGR samples were divided into two major clusters and the variation in their color provided a good indication of the differences in their quality. Notably, we observed a statistically significant correlation between the color metrics and the quality of the different SGR samples (Pearson’s r = −0.769, *P* < 0.001).

## Abbreviations

CM: Chinese medicine
SGR: *Smilacis Glabrae Rhizoma*
R-SGR: red SGR
W-SGR: white SGR
RGB: Red–Green–Blue
UPLC-Q-TOF-MS: ultra-performance liquid chromatography-quadrupole time-of-flight mass spectrometry
CLA: color A
CLB: color B
CHA: chemical A
CHB: chemical B
ESI: electrospray ionization
EIC: extracted ion chromatogram
RPA: relative peak area
RT: retention time
IS: internal standard
KMO: Kaiser–Meyer–Olkin
RSD: relative standard deviation
RRT: relative retention time
